# Creating work-life balance among physicians in the age of digitalization: the role of self-consciousness and communication – a qualitative study

**DOI:** 10.1186/s12913-023-10101-w

**Published:** 2023-10-24

**Authors:** Zsuzsa Győrffy, Nóra Radó, Lea Pölczman, Anikó Sükösd, Julianna Boros

**Affiliations:** 1 Institute of Behavioral Sciences, Faculty of Medicine, Semmelweis University, Nagyvárad tér 4. 20th floor, Budapest, H-1089 Hungary; 2 Eötvös Lorand University, Pázmány Péter sétány 1/A, Budapest, H-1117 Hungary; 3 Hungarian Demographic Research Institute, Budapest, Hungary

**Keywords:** Work-life balance, Digital health, Physicians, Blurring boundaries, COVID-19, Burnout

## Abstract

**Background:**

Besides the positive effects of using digital health solutions, digitalization can affect the healthcare worker burnout. The ability to coordinate different aspects of life (WLB) also plays a significant role in the development of burnout among medical workers. The aim of our study is to show, through qualitative interviews, the impact of digitalization on work-life balance in Hungarian physicians.

**Methods:**

62 semi-structured interviews were conducted between October 2021 and June 2022, of which, a total of 31 interviews were used for the analysis, which were all related to the theme of work-life balance. Purposive sampling and inductive thematic approach were used to collect and analyse the data and identify patterns of the themes.

**Results:**

Based on this analysis, 5 main themes emerged: (1) the use of digital health tools, (2) Impact of digital tools on everyday work, (3) Work-life balance, (4) Setting and maintaining work-life boundaries, (5) Potential solutions. With the spread of digital communication, most of the respondents feel that their working hours increased even at the expense of their private life. The majority considers constant availability as a serious problem, however, several physicians indicated that as a result of a learning curve, they are able to change and set the necessary boundaries. Respondents were divided on whether or not they were successful in setting and maintaining boundaries. The 2 most important factors of establishing WLB in a digital age are self-consciousness and communication. However, these skills are not self-evident: the responses also show that in many cases there is a need for external support, but also for health professionals to actively reflect from time to time on their role as healers and their relationship with technology.

**Conclusion:**

Basic principles and tools for establishing successful digital work-life balance in healthcare should be involved in the training curriculum of future physicians and healthcare professionals, while institutions should elaborate specific policies to include digital work-life balance in the institutional setting, as part of the preventive measures against burnout.

**Supplementary Information:**

The online version contains supplementary material available at 10.1186/s12913-023-10101-w.

## Introduction

A number of studies confirm the huge benefits of different digital technologies in everyday patient care: convenience, speed, efficiency and the ability to involve patients better in the treatment process [1]. At the same time, digital health solutions still increase the administrative burden on physicians and working hours in a lot of cases [2].

The effects of digital health solutions on physicians’ working conditions and well-being have been well-researched even before the COVID-19 pandemic. In these studies, the effect of digital administration (EHR, Epic) and that of working with actual digital technologies are separated. While the former has been shown in many studies to be a trigger for burnout, the use of various digital devices has been shown in many cases to increase work efficiency and job satisfaction, as a factor specifically acting against burnout, although the connection is not always clear [3]. The systematic review of Yan et al. did not show a direct connection between burnout and digital administration [4]. On the other hand, Ghatnekar et al. in their literature review found that the introduction of a digital scribe can act specifically protective with regard to burnout. Digital scribe technologies can improve clinic efficiency and increase patient access to care while simultaneously reducing physician burnout [5, 6]. Golz et al. found in their study examining psychiatric care workers that workers who are in intensive contact with digital devices self-evaluated their technostress higher and their digital competencies lower than those who did not work with such devices frequently [7]. When examining other aspects of being digitally present, according to the results of several studies, continuous online presence, social media and screen use, as well as patient feedback platforms could increase burnout [8–10].

Despite a few research projects and the fact that the problem of medical worker’ burnout become one of the key issues of the COVID-19 pandemic, there is a relative lack of studies that research on the role of digitalization in medical worker’ burnout. The terms of technostress and e-stress already appeared way before the pandemic at the advent of digitalization [11–13]. Its symptoms include distraction, reduced concentration, anxiety, fear, and general physical symptoms [14]. The root causes could include technical difficulties (“computer freeze”), overstimulation by using too many types of devices, information overload, as well as multitasking. Intensive technology use could increase workload and blur the boundaries of work and private life disappear, and all of this could have an impact on medical worker burnout [15]. During the COVID-19 pandemic, the term “telepressure” became widespread, which means the pressure to respond to emails and other messages coming through different channels without any delay [16]. Kasemy et al. surveyed Egyptian healthcare workers during the COVID-19 pandemic, and highlighted the associations between technostress and burnout, as well as that of technostress, high cortisol levels and low job satisfaction [17].

In addition to digitalization, the ability to coordinate different aspects of life (Work-Life Balance, WLB) also plays a significant role in the development of burnout among healthcare workers, as several previous studies have already pointed out [18–21]. The integrated model of Carlson et al. [22] identified different types of work-life conflict: time-based (arising from lack of time), stress-based (arising from role-mismatch stress), and behavioural-based (arising from different behavioural expectations). These studies showed that difficulties arising from the need to coordinate work and family life have an impact on job satisfaction [23], and physical and mental health [24, 25] Several studies add that WLB impacts vital exhaustion, depression, and burnout [26–28]. There is a significant negative correlation between WLB and the emotional exhaustion (EE) and the depersonalisation (DP) subscale of burnout [29].

Recently, theories on achieving work-life balance (WLB) have gained prominence. These theories emphasize the importance of individuals being equally engaged and satisfied with their roles in both work and family life [30]. They suggest that work and family domains should align effectively, allowing individuals to succeed in both areas. This involves ensuring that resources from one domain are in line with the needs of the other domain, creating a harmonious balance [31, 32].

As digitalization permeates both domains of work and family life, it also gives a new dimension to WLB. Related to this, the concept of Digital Life Balance (DLB) was born which means the successful harmonization of online and offline life [33]. The digitalization of healthcare challenges the successful coordination of work and private life - in terms of working hours, availability, as well as keeping boundaries.

The concept of “digital health” is difficult to define precisely. Fatehi et al. in 2020, after reviewing nearly 1500 articles, identified about 95 types of scientific and “everyday” definitions [34]. Some definitions emphasise the technological aspects of the topic (digital health and health care, for example refers to mHealth, telehealth, telemedicine, wearable devices), while others emphasise the human component: personalisation, the transformation of the doctor-patient relationship, the democratisation of health care [35]. In this paper, we explore the usage and cultural changes associated with digital health. In this conceptual framework, digital health is not only a technical and technological revolution, but also a cultural and social transformation: the transformation of the doctor-patient relationship, decision-making and health management. [35].

In our exploratory research undertaken in 2021–2022, we focused on the topic of digitalization and WLB. In this qualitative analysis, we aimed to analyse what impacts digital communication, digital administration, and the use of digital tools could have on the ability of individuals to reconcile work and family life, and what kind of solutions surface when striving for WLB in Hungary.

## Methods

Within the framework of the “E-patients and e-physicians in Hungary: The role and opportunities of digital health solutions in the healthcare system” (OTKA-FK 134,372.) research program, a semi-structured qualitative survey was conducted, involving 62 interviewees. Our study is based on the *consolidated criteria for reporting qualitative research*(COREQ) checklist [36] (see Supplementary Material 1). Written informed consent statements were obtained in all cases, and ethics approval for the study was issued under TUKEB:133/2020 and IV/10,927/2020/EKU by the Scientific Research Ethics Committee of the Medical Research Council of Hungary.

### Sample

Purposive sampling was based on the following criteria: (1) physicians who are actively involved in patient care, (2) work in Hungary and (3) have experience in digital health solutions. The information power criteria based on [37] were (a) the aim of the study, (b) sample specificity, (c) the use of established theory, (d) quality of dialogue, and (e) analysis strategy. Since the aim of our study was to assess different aspects of digital transformation (see Supplementary Material 2 for interview guide) in the medical profession and our analytical framework was thematic analysis, the larger sample size was chosen. The research was completed with 62 interviews. In the present analysis, we worked with interviews in which the theme of work-life balance appeared (n = 31).

### Data collection

The one-to-one, semi-structured interviews were conducted between October 2021 and June 2022. At the time of the research, Hungary had already passed the third wave of COVID-19, which was much more serious than the previous ones in terms of both the number of illnesses and deaths, reaching outstanding values even in international comparison. However in autumn 2022, due to the omicron variant, the number of cases started to rise again, so several government measures were introduced and re-introduced (compulsory vaccination in certain jobs, compulsory mask-wearing, ban on visits to health facilities). The epidemic situation has had a strong impact on the daily work of healthcare workers, causing significant changes compared to the period before COVID-19. For the purposes of our research, we define telemedicine as the following: it is a health service where the person receiving care and the person providing care do not meet directly, but the connection between them is established through a remote data transmission system. In most cases, it means that healthcare is moving partly or entirely to the online space but according to the Hungarian protocols, telemedicine also contains telephone medical consultations.

The interview guide was developed from the study aims and literature review. The interviews were conducted in Hungarian with trained interviewers, the interview guide was checked on a physician sample (n = 4) and modified based on their feedback. The interview guide is based on the following topics: work and career choice, technological changes in the medical field over the past decades, the different types of digital health devices and services they use/know, how the doctor-patient relationship changed since the start of their career, and what they think about the future role of digital health (See Supplementary Material 1 for the complete interview guide).

Interviews were audio recorded in person and online (zoom video call), with an average interview length of 60 min. All audio recorded interviews were transcribed verbatim and each transcript was anonymized and attributed a unique code. The interviewers checked the transcriptions for accuracy. Then, the final transcripts were sent back to each interviewee for confirmation and feedback.

### Analyses

The theoretical framework for the analyses was thematic analysis created by Braun and Clarke [38]. In coding, we followed the inductive technique, i.e. we did not work with predetermined assumptions and themes, but tried to identify the contexts that emerged when reading the transcripts. Five independent researchers (Zs.Gy., J.B., L.P., N.R., A.S.) read and analysed the data and discussed their findings.

We used an inductive thematic approach to analyse the data and identify patterns of themes: (1) familiarizing with the content of the data and taking notes and making ideas for coding, (2) generating initial codes, (3) identifying and indexing different codes across the data set, (4) reviewing themes creating relationships between the themes and subthemes, (5) defining, mapping and naming themes and (6) interpreting our results. The 5 researchers discussed and developed all themes and subthemes and clarified any discrepancies during the coding. After then they evolved the final thematic map, which was laid down in mutual agreement. Our results were supported by anonymized quotes from different participants. All interviews were coded using Atlas.ti 6.0. software.

**Fig. 1:**
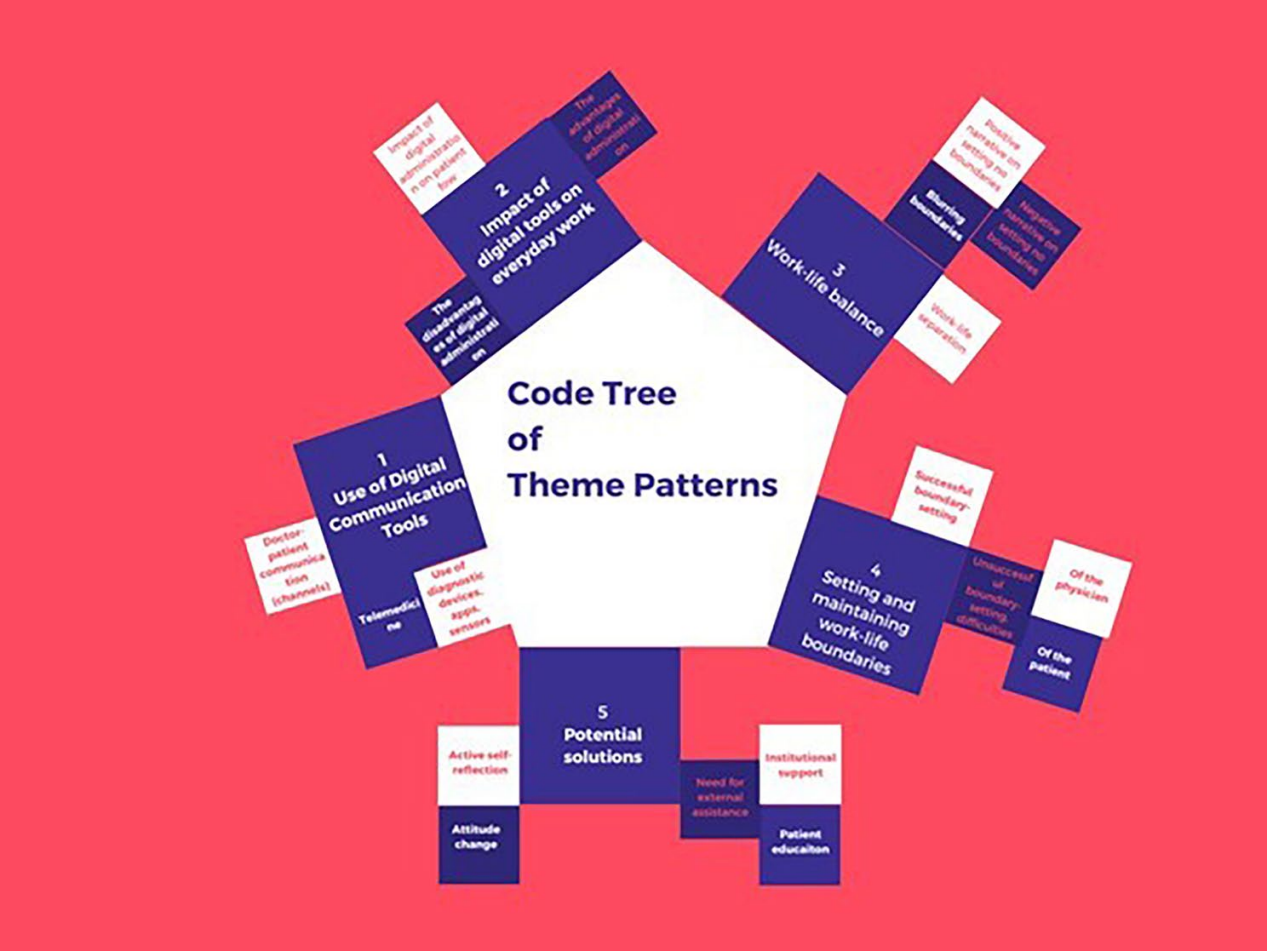
Code tree of theme patterns

For an overview of the themes see Fig. 1.

## Results

### Demographic characteristics of the sample

A total of 31 interviews were used for the analysis, all of which were related to the theme of work-life balance. The interviewees included 17 women and 14 men. In terms of age, 14 were in the youngest category, under 40, 15 were in the 40–59 age group and 2 were over 60. By municipality, 17 worked in Budapest, 4 in the county seats, 8 in other towns, 1 in a village and 1 abroad. Out of the doctors who participated in the survey, 14 worked in primary care, 10 in inpatient care and 7 in outpatient care.

## Theme 1: use of digital communication tools

Three subthemes emerged from the topic of digital communications tools: channels of doctor-patient communication, the use of telemedicine options, and the use of digital health devices.

All the interviewees had their own experiences with digital administration or digital communication tools or digital health devices (applications, sensors). It could be discerned from the interviews that they all use EESZT (National e-Health Infrastructure), online training options, online literature search, and online consultation with fellow physicians.

### Doctor-patient communication (channels)

Independent from the area of expertise, communication via traditional phone lines are the most frequent method beyond personal doctor-patient communication and offline patient screening. This communication channel is prevalent in every interview, except in diagnostic areas of expertise (e.g. radiology), where doctor-patient communication is not part of the workflow. All of the interviewed physicians use email communication, and several of them mentioned that they have their own information and/or education platform (Facebook page for the practice, Facebook group, own blog). However, there is a huge difference between the communication channels of physicians working in primary and specialized care: family physicians typically use several channels - beyond traditional phone-based communication.

### Telemedicine

During the COVID-19 pandemic, practically every single interviewee had phone visits, used e-prescriptions, transferred medical records online and engaged with online scheduling systems. The last three remained a significant part of their jobs ever since.

*“There is the telemedicine option, which means when a patient calls and tells me [their problem], and I write it on the outpatient card, they don’t even have to come in, it will get into the system. You don’t need to use email which is not that formal. And then, for example, the EESZT is also very, very good for writing prescriptions.*” (Demo-19).

However, now personal patient visits with actual patient screenings dominate when it comes to acute care. Only 5 interviewees reported that they have already tried telehealth video visits, and they all work in primary care.

### Use of diagnostic devices, applications, sensors

Users of diagnostic devices, applications, and sensors are typically members of the younger generation and those who are more digitally open use more tools both with their patients and in their private life. Transtelephonic ECG is an important telediagnostic device, which is used by most physicians working in a GP’s office. In one or two cases, the use of risk assessment apps (deep vein thrombosis, pulmonary embolism risk assessment apps) was mentioned, in one interview, a physician mentioned the pilot application of virtual reality glasses in pediatric oncology, in another a digital stethoscope in paediatrics, and an otoscope or a fiberscope in ENT was listed.

*“I have a digital stethoscope, an otoscope […] I can take photos, analyse them and send them to the specialist to help his work. We also have a transtelephonic ECG, the results of which can be analysed by a professional cardiologist, and sent back almost immediately after evaluation. For me, UH diagnostics would also be important, it is not necessarily an issue of financial nature, it’s more about training opportunities.”* (Demo-30).

## Theme 2 impact of digital tools on everyday work

Three subthemes emerged when we examined the topic of using digital tools and their administrative impact on everyday work: the advantages and disadvantages of digital administration, and the impact of digital administration on patient flow.

Every interviewee experienced the impact of the pandemic on online communication. And several of them mention that with the spread of digital communication, their working hours were extended even at the expense of their private life. On the other hand, administration due to technological changes has multiplied, which increases working hours.

### The advantages of digital administration

As advantages of digital administration, respondents mentioned that e-prescription makes the physical patient-doctor meeting redundant, that the records from doctors in charge became available, and that the already available option of accessing medical records remotely increases virtual mobility. The latter makes it possible for hospitals to help each other out.

### The disadvantages of digital administration

One interviewed physician mentioned the general demand of patients for the physician to send everything to them via email as a problem. It generates more work for them, and the doctor-patient communication will become more difficult. According to another respondent, technological tools and software became so complex that one simple task takes 3–4 times more time longer than before. A specialist working in paediatrics said that as paper-based documentation is still required, both digital and paper-based documentation is carried out at the moment, which is “a bit too much”. Furthermore, it is mentioned as a problem that patients tend to write very long emails even with regard to very small issues; thus, managing emails is a daunting task.

*“Patients usually send emails for prescriptions, and I respond sooner or later, but this takes up quite a lot of time. We are always short of time, I only use emails because I can check them whenever I want, and I only respond whenever I want or can and I have the time and energy for it. I don’t use faster online platforms, because the sheer amount of queries and incoming information is so huge that it has to be toned down somehow.” (Demo-17)*

### The impact of digital administration on patient flow

One of the interviewees expressed their hope that digitalization will speed up follow-up patient visits. Another respondent highlighted that the role of telemedicine could be recognized through its capacity to decrease workload and not in taking over the role of traditional medicine. Also, the introduction of the online scheduling system has made the day-to-day running of the practice much more coordinated and predictable. Although some interviewees reported that email and phone consultations do reduce patient volumes, as mentioned above, dealing with and responding to emails is a major challenge for physicians.

All of the physicians indicated that the COVID-19 pandemic has led to an increase in queries in the online sphere and that the option of writing e-prescriptions was frequently used from home. Several interviewees mentioned that they responded to incoming messages on Messenger and their Facebook page during the pandemic, moreover, they specifically asked their patients to report back to the physician about their state also via email and SMS. Now, on the other hand, they do not use these channels anymore.

## Theme 3 work-life balance

In our analysis, the theme of work-life balance was divided into two subthemes: blurring boundaries and work-life separation. The majority of the interviews contain a reference to constant availability as a serious problem, however, several physicians indicated that as time went by, they had the ability to change and set up the necessary boundaries as a result of a certain learning curve.

### Blurring boundaries

Blurring boundaries can mean the constant availability of doctors, constant communication with patients (emailing, scheduling, etc.), and no separation of working hours and free time. This can have both positive and negative narratives on the part of doctors.

#### Positive narrative on setting no boundaries

Many of the interviewees explained their positive attitude by their sense of vocation, their empathy towards patients, and the fact that they feel they can always be counted on.

*“The difficult thing is that the holidays and the weekends completely overlap… I’m not good at separating work and my private life, but I don’t even try to because I know that it’s a great help to my patients if I’m available.” (Demo-55)*.

Some reported that even in the case of their own illness or other limiting factors, they are still available to their patients through applications and programs available at home, so their sense of responsibility towards patients overrides their health-related disability.

#### Negative narrative on setting no boundaries

However, there is also a fair number of physicians who consider blurring boundaries of work and private life as a negative phenomenon, perceiving it as a burden. Several interviewees highlighted that an unmanageable amount of work has ended up on their hands. Some are frustrated by the constant pressure to perform both well in their work and their family life. A couple of physicians said that they found it difficult to meet the expectations of patients and their families, and to maintain a healthy work-life balance. Many physicians reported that it is difficult to get off work due to the nature of therapeutic work in the first place. Around half of the interviewees said that it is difficult for them to relax after working hours and let go of work-related rumination. An obstetrics and gynaecology resident articulated this in the following way:

*“It doesn’t matter whether we go home after work, we still often think about the patient and what we could have done differently, how we could have helped better.“ (Demo-46)*.

One of the interviewees pointed out that the mixture of constant preparedness, therapeutic work and digital communication could lead to a decrease in quality, as an increase in quantity leads to a decrease in quality. But it can also make concentrating difficult, which also has an impact on the quality of their work.

*“A disadvantage of digital communication is that it distracts you. I’m concentrating on a patient, I try to have a conversation, but the phone rings, a new notification appears on the computer screen, there’s a new message, and someone wants to get an appointment. All of this scatters the attention, you cannot pay attention to the patient. I’m truly not a multi-tasker, but I don’t think anyone is. You have to filter that huge amount of incoming information. You cannot do everything at the same time, pay attention to everyone.” (Demo-17)*.

### Work-life separation

Work-life separation does not appear to be a problem for physicians in some cases. This can arise from the nature of the work, as in the case of several specialities, digital doctor-patient communication is unrealistic. A surgeon, a paramedic and a radiologist all reported that this is not part of their medical work.

In some cases, that the lack of digital tools results in work-life separation. This can happen due to the physician but this is a rarer case. It is more typical of physicians of older age.

## Theme 4 setting and maintaining work-life boundaries

The efforts to achieve work-life separation and balance appeared in every single interview. In this theme, two subthemes emerged: successful and unsuccessful boundary-setting, and difficulties.

### Successful boundary-setting

Those physicians, who reported successful boundary-setting, could be characterized by the setup of time frames and appropriate time management. Some of these physicians do not constantly provide online communication, emailing, or phone services, and they set up an exact time frame for such services. Some of them do not respond to incoming emails on the weekend, only during working hours. Some physicians set up time-based boundaries: they tell patients when to write messages to the social media page of the general practice. Doctors who can maintain their boundaries, and those who provide digital services only during face-to-face care, said that it was important to them that patients could not contact them outside of working hours and work, otherwise it could have been a burden on their private lives. A primary care physician put it this way:

*“I could work through my entire day if I wanted to… work never ends, and me, my assistant, and my colleague, we all experience that. This is difficult, that’s why […] we also have to set up our boundaries in a clever way.” (Demo-27)*.

It is also a noticeable trend that interviewees started to limit their online presence consciously. Those who do use social media try to maintain the boundaries of work and private life also in the online sphere. A physician reported that they communicate with their patients exclusively on the official social media page of the general practice and they manage their own user profile separately. Another interviewee said that they would not install any work-related applications on their private mobile phone; and another physician confirmed that they are only using social media in their private life.

### Unsuccessful boundary-setting, difficulties

Behind unsuccessful boundary-setting and difficulties maintaining the boundaries were problems on the part of the doctor and perceived difficulties on the part of the patients.

#### Unsuccessful boundary-setting of the physician

Physicians experienced unsuccessful boundary-setting and difficulties due to their own expectations towards themselves most of the time. A particular difficulty is that many doctors do not set clear boundaries and limits for themselves. Unfinished tasks, such as answering an email, are often difficult for them to keep in mind and they feel overwhelmed by a sense of urgency. This is why they often answer their patients’ messages on weekends, as they find it hard not to respond.

*“It happens to me as well that the email window of the practice stays open as a tab in the browser, and then I sometimes look at it involuntarily even on weekends.”* (Demo-41).

#### Unsuccessful boundary-setting of the patient

All the doctors interviewed experienced difficulties from patients, which made it difficult to maintain a work-life balance. One problem was non-compliance with the time frames on the part of the patients. The majority of the physicians reported that patients had come forward with their cases after the physicians’ office hours at the expense of their free time. Many patients write messages or emails to their doctors even on weekends, holidays, or at night. Some interviewees complained that patients have a difficult time figuring out in what kind of cases it is reasonable to use the digital services of a physician outside of working hours. For most of the respondents, it also meant a further difficulty that some patients contact them with inappropriate attitudes, without respecting the basic rules of politeness when using online communication. Moreover, physicians also found it the difficult to choose the appropriate communication channel: sometimes patients cannot differentiate between a private or a general practice’s user profile. Almost half of the interviewees expressed that patients often leave the private life of physicians out of consideration. Moreover, half of the physicians have the sense that patients expect them to be available all the time. By using a social media site, they are also provided with the platform or the option at least.

## Theme 5 potential solutions

The interviews reveal that every single physician had some kind of a solution for the challenges arising from the use of digital communication tools primarily regarding the pressure on work-life balance. From the interviews, it is clear that physicians’ patterns of coping can be divided into three groups: a small number of them are conscious, i.e., they take concrete steps to set up and consistently respect their boundaries in work and private life also online sphere, another small number of them could be characterized by a certain attitude change, i.e. they mitigate the pressure by letting go of their boundaries, extending their online availability and focusing on patient satisfaction.

The majority of the physicians, however, do not show any signs of elaborate solutions, but the initiative and the need for change are already there, and they also formulated ideas on what and how to change. This need appears in two ways: either by articulating the need for patient education or the need for institutional support, i.e. in the form of training for physicians.

### Active self-reflection

A small number of the interviews reveal good practices that can help alleviate the stress of meeting patient expectations - such as being available all the time as a doctor - and the resulting pressure on how to maintain a work-life balance. One is to actively keep in mind that setting up and maintaining boundaries is a means to relieve pressure and it is worth applying some practices that can help maintain those boundaries.

This could mean the conscious use of digital tools, or setting the limits of online presence: it could mean defining time frames for email communication, for example, differentiating between weekdays and weekends, between working hours and rest periods in terms of online availability; for social media sites, separating official and private pages; rejecting requests and messages on private pages; and it could also mean using a mobile application on a digital device dedicated for work and only receiving digital enquiries from patients through that device, but in case without having a dedicated work-phone, not installing work-related applications on one’s private device.

A common characteristic of physicians showing signs of active self-reflection is that they changed their initial usage patterns, and the exceptional period of the pandemic also shaped their digital habits.

*“I also experience it very frequently that a mom sends me a Facebook friend request, and then I consciously choose not to befriend her because they are not my friends, they are not my acquaintances and they don’t have to be, and there’s also no need for me to be constantly available for them, because say they would send me a picture that there is something on the child’s bottom and I shall look at what that is - but what if I’m at home having dinner with my family?” (Demo-18)*.

#### Attitude change

The other solution, in contrast to the describe above is to be aware that blurring the boundaries is the solution to relieve pressure, since meeting patients’ requests and expectations is part of the medical profession, and patient satisfaction brings physician satisfaction. This unique attitude was prevalent in two interviews. Through this attitude change, the blurring of boundaries between work and private life receives a positive association instead of a negative one.

*“About telephone communication beyond working hours: well, those who believe that’s a burden should not choose this profession because, for such a person, work will not bring any joy.” (Demo-18)*.

### Need for external assistance

In addition to individual solutions, the need for external help to overcome the challenges posed by digital tools is also a major subtheme in the interviews. This means both education and the adaptation of the institutional environment to the changed circumstances.

#### Patient education

When educating patients, some doctors recognize their own responsibility and the importance of informing their patients about i.e. how their work schedules will look in the age of constant online presence, while others see the responsibility to use digital tools and manage online availability as part of a certain general digital preparedness on the part of the patients. The education of doctors also appears at the individual level, as self-training, and also in institutionalised form, for example when an interviewee talked about having a “good system” in place.

*“It’s also important that the patient feels how long an email can be, what kind of response they expect, and that this shouldn’t take up my free time, because for the time being it’s all I have time for.” (Demo-47)*.

#### Institutional support

In some of the interviews, there is a need for an institutional response to regulate the use of digital tools, for example, to respond to requests by email or social media sites during working hours, or that the hospital or any other healthcare institution should fund a tool that can be used specifically to communicate with patients.

A number of interviewees expressed that digital communication did not officially become part of working hours; this task is completed on the expense of their own free time. Some highlighted that if digital communication (emailing patients) happened during office hours, then they would not have the possibility to perform actual therapeutic activities.

*“I would have to schedule appointments for so many people that if I responded to everyone, I wouldn’t be able to examine the patient sitting in front of me.” (Demo-17)*.

One of the interviewees said that digital workload becomes a burden when they go on leave as replacement only involves personal patient care. Digital communication is excluded so they have to do it themselves even during leaves.

Some people expressed that there was no financial compensation for the overtime work, as telemedicine done in their spare time is not compensated for: the extra work over and above the required working hours is essentially done for free. As a potential solution, institutions might consider incorporating all this extra work into the official workflow. Moreover, dedicated digital assistants, language processing algorithms and chatbots could be a viable institutional strategy to alleviate the burden on physicians and give them a chance to estabilish a viable WLB.

## Discussion

The COVID-19 pandemic played a crucial role in the digitalization of healthcare, and technological changes brought about many further alterations to numerous aspects of work. Although the pandemic catalysed the spread of digital health solutions in an extreme way, competent usage lags behind both on the patient’s and the provider’s part [39]. Beyond positive changes, problems of “technostress”, “digital burnout”, and time pressure are also prevalent. Several studies showed that beyond undeniable advantages, we have to deal with the following questions as well: the development of digital competencies, setting up and maintaining boundaries of work and private life, the avoidance of the depersonalization of the doctor-patient relationship, the adaptability of digital tools to working hours, the problems of financing, as well as connecting the physical and digital worlds in a viable way. These have become very important questions requiring future solutions [9, 40, 41].

Technological transformation, digitalization and home office as a new way of work are double-edged swords for not only healthcare related jobs but in general as well, as on one hand they bring flexibility, autonomy and greater productivity [42]; while on the other hand, they make it difficult to successfully coordinate different aspects of life. For healthcare workers, successfully reconciling work and private life has always been a significant challenge but it has been further exacerbated by digitization and the COVID-19 pandemic [43]. Digitalization redefined the question of WLB - in healthcare as well, and it has had a different impact on different countries. In highly digitalized countries where the digital transformation of healthcare was completed before the COVID-19 pandemic, further digitalization could have a positive impact on WLB but in countries with a low extent of digitalization, where the shock of the pandemic went hand in hand with the rapid introduction of digitalization in healthcare services - i.e. in the Central and Eastern European countries, such as Hungary -, this phenomenon could apply greater pressure on WLB [44, 45].

According to Zaresani and Scott’s study based on the analysis among Australian physicians about the association between using digital health technology and the probability of reporting high job satisfaction and a good work-life balance, physicians who used digital health technology were 14.2% points and 20.3% points more likely to report respectively higher job satisfaction and good work-life balance, compared to the physicians who did not use it [46].

Nevertheless, the COVID-pandemic exerted such a shock on healthcare systems in Europe, in the Americas and Asia that even highly developed systems had to face serious challenges of being able to provide appropriate patient care, not to speak about WLB. Bakhai et al. reported that COVID-19 increased the stress of NHS workers in the UK by making them unable to separate digital work communications from family and home time. A large proportion (36%) of staff had already been unable to switch off from work-related communications before COVID-19, which worsened (57%) during the pandemic [47].

In line with the conclusions of major international research projects, the results of our qualitative research show that all of the interviewed physicians experienced the difficulties in achieving work-life balance. These were due to the specific situation created by the COVID-19 pandemic, the particular difficulties of digital communication and administration, and the rapid introduction of digital tools into everyday medical practice during the very beginning of the pandemic, where previous knowledge and use of these tools was minimal.

The lack of a clear definition of how digital communication and administration can be integrated into everyday medical work creates further difficulties. In the majority of the interviews, it appears that the constant availability that comes with digitalization is a serious challenge, but various narratives also presented that a learning process and choosing a potential solution - either consciously choosing constant availability or setting up boundaries - can help alleviate the stress that comes with it.

In the medical profession, maintaining boundaries (availability, constant mental presence, ruminating on problems) is not an easy task. This difficulty has now been extended by the fact that technology allows anyone to be present and work all the time. When it comes to the creation of WLB and maintaining boundaries, those interviewees were the most successful who either consciously kept no boundaries between the two areas or consciously made efforts to set up and maintain boundaries between work and family life, and they communicated it towards themselves and their patients successfully. Our qualitative research based on interviews also showed that the creation of WLB is the result of a relatively slow process. It is common among the physicians who showed signs of active self-reflection that they changed their initial usage patterns and they shaped their digital routines in terms of their experiences.

According to our research, it can be stated that the two most important pillars of the creation of WLB in a digital age are self-consciousness and communication. However, these skills are not self-evident: the responses also show that in many cases there is a need for external (even institutional) support. At the same time health professionals also need to actively reflect from time to time on their role as healers and their relationship with technology. Both in the education and training of healthcare professionals and in patient education, it would be important to establish clear rules, to deepen the ways and possibilities of digital communication, and to review the relationship with digital technology from time to time. For this, the Digital Skills Assessment Tool [48] could be a great help for healthcare professionals working in the system of the British National Health Service (NHS). It determines the current digital literacy levels of the user, and helps identify areas of learning need, then redirects the user to learning resources.

However, in order to reduce technostress, it is also important that healthcare workers become more proficient in the world of digital technology - so in addition to soft skills (e.g. standards for digital communication), education on the use of digital tools and programs would be necessary.

In the interviews, however, the need to educate not only health professionals but also patients were raised: patient education was understood as an obligation of the physician, the hospital/medical practice and society as a whole, but the “host” of this educational task was not specified by the interviewees. Other examples show that digitally engaged doctors are embracing patient education and that some institutions are trying to provide digital guidance to patients [49]. For example, Leeds University Institute of Medical Education has a Patient Carer Community where medical students teach digital skills to patients, for example, and in this way, medical students also get feedback on what patients really need, what and how to communicate to them, what communication channel to choose and how WLB can work effectively [50].

While the digital working schedule during the pandemic had been challenging, various digital solutions (BurnOut App) might have been an important support in maintaining mental health for healthcare workers [51–56]. Adequate technical support, increased training and digital competencies, optimisation of working time, flexibility and a greater sense of control over work reduce the “technostress” experienced by doctors [40, 57–60].

The strength of our research is that relatively few studies have focused on work-life balance and digitalization issues among doctors. It is both a strength and a weakness that the interviews were conducted during waves 3 and 4 of the COVID19 pandemic. The pandemic and associated patient care protocols influenced the response, therefore the results are not automatically transferable to post-COVID times - although the pandemic has undoubtedly changed digital health habits, so once the threat has passed, it is not back to business as usual. Another limitation of the study stems from the sampling principles: we sought to interview people with considerable experience in the world of digital health, so those who were less committed to using digital technologies were excluded, e.g. the number of senior physicians was relatively small among the interviewees. Furthermore, due to the nature of the study, burnout and work-life balance were assessed using non-standardized questionnaires. Our study could be an important starting point for further quantitative studies; however the qualitative approach of the study does not allow for generalisation of the results.

## Conclusion

How to draw the line between work and private life for healthcare personnel is a constantly re-emerging research topic as the appearance of new factors re-write the delicate work-life balance again and again. Such a novel factor is the digitalization of healthcare, as it had overturned among others the system of administration and written and unwritten rules of communication between doctors and patients.

It has been happening differently in countries with different levels and speeds of healthcare technology adoption, however, there seems to become a consensus in the field that the COVID pandemic has accelerated technology adoption on a global level. This research was undertaken during the pandemic, in a period with intense workload and pressure on healthcare workers, which also has its imprint on our findings.

The 31 interviews showed how Hungarian healthcare workers had been dealing with the first shock of the COVID pandemic as well as that of the digital work schedule, how the high-intensity periods of the pandemic rotated and how the workload alongside with the pandemic subsided. The interviews revealed how physicians reflected on their workloads, how they tried to adjust their work and private lives to the altered situations, and how they re-arranged their work-life balance again after the intense periods of the pandemic. Although we examined the Hungarian situation, many other countries had to face very similar circumstances, and are still searching for their way of a successful transition towards 21st-century healthcare.

This learning curve has valuable lessons to be drawn so that the healthcare workforce can be prepared for the challenges of the future more successfully. As the digitalization of healthcare is not going to slow down, the education and training system as well as the health institutional settings should involve and reflect on the digital component more actively. Basic principles and tools for how to set up a successful digital work-life balance in healthcare should be involved in the training curriculum of future physicians, and healthcare professionals, while institutions should elaborate specific policies to include digital work-life balance more in the institutional setting, as part of the preventive measures against burnout.

However, the setup, introduction, and education of a digital health policy that incorporates digital work-life balance might not only help prevent healthcare workers on the individual and the institutional level, but also on the macro level: it would increase the resilience of the entire healthcare system.

### Electronic supplementary material

Below is the link to the electronic supplementary material.

Supplementary Material 1

Supplementary Material 2

## Data Availability

The datasets used and/or analysed during the current study available from the corresponding author on reasonable request.

## List of Abbreviations

DP: Depersonalisation
EE: Emotional exhaustion
EHR: Electronic Health Record
DLB: Digital Life Balance
NHS: British National Health Service
WLB: Work-Life Balance
