# Supplementary material for: Creating work-life balance among physicians in the age of digitalization: the role of self-consciousness and communication – a qualitative study
Source: BMC Health Serv Res. 2023 Oct 24;23:1141. doi: 10.1186/s12913-023-10101-w (PMC10594792; doi:10.1186/s12913-023-10101-w)
Supplement: Supplementary file 1 — Supplementary Material 1 [file 12913_2023_10101_MOESM1_ESM.docx]

**Completed checklist of the consolidated criteria for reporting qualitative research (COREQ)^1^**

| **No. Item** | **Description** |
| --- | --- |
| **Domain 1: Research team and reﬂexivity** |  |
| *Personal Characteristics* |  |
| 1. Facilitator | Interviews were conducted by professional interviewers (2 people) |
| 2. Credentials | MA |
| 3. Occupation | Sociologist |
| 4. Gender | Male and female |
| 5. Experience and training | Trained interviewers; several years of experience in fieldwork |
| *Relationship with participants* |  |
| 6. Relationship established | None (interviewees were unknown to the interviewers) |
| 7. Participant knowledge of the interviewer | Interviewees were informed in a written form in advance of the purpose of the interview |
| 8. Interviewer characteristics | Interests in the research topic |
| **Domain 2: study design** |  |
| *Theoretical framework* |  |
| 9. Methodological orientation and Theory | Thematic analyses |
| *Participant selection* |  |
| 10. Sampling | Purposive sample |
| 11. Method of approach | Face-to face, but due to COVID19 pandemic most interviews were conducted via video call; the interviewers have made a prior telephone/email appointment with the interviewees |
| 12. Sample size | 62 |
| 13. Non-participation | 5 people refused to participate due to scheduling problems |
| *Setting* |  |
| 14. Setting of data collection | Home and workplace |
| 15. Presence of non-participants | Only the participants and the interviewers were present |
| 16. Description of sample | Physicians sample, the interviews were taken during the COVID-19 pandemic  A total of 31 interviews were used for the analysis, all of which were related to the theme of work-life balance.  The interviewees included 17 women and 14 men. In terms of age, 14 were in the youngest category, under 40, 15 in the 40-59 age group and 2 were over 60. By municipality, 17 worked in Budapest, 4 in the county seat, 8 in other towns, 1 in a village and 1 abroad. Of the doctors who participated in the survey, 14 worked in primary care, 10 in inpatient care and 7 in outpatient care |
| *Data collection* |  |
| 17. Interview guide | 4 pilot interview conducted. The topic guide was finalized based on the feedbacks, |
| 18. Repeat interviews | N/A |
| 19. Audio/visual recording | Audio recording and transcription |
| 20. Field notes | No field notes at the setting; ex-post notes about the circumstances of the interviews |
| 21. Duration | Average interview length: 60 minutes |
| 22. Data saturation | No |
| 23. Transcripts returned | yes |
| **Domain 3: analysis and ﬁndings** |  |
| *Data analysis* |  |
| 24. Number of data coders | 5 |
| 25. Description of the coding tree | See Figure1 |
| 26. Derivation of themes | Inductive thematic approach |
| 27. Software | Atlas.ti 6.0. |
| 28. Participant checking | no |
| *Reporting* |  |
| 29. Quotations presented | Participant quotations are presented to illustrate the themes/ﬁndings; each quotation is identiﬁed with interview number |
| 30. Data and ﬁndings consistent | yes |
| 31. Clarity of major themes | yes |
| 32. Clarity of minor themes | yes |

1. *Tong A, Sainsbury P, Craig J. Consolidated criteria for reporting qualitative research (COREQ): a 32-item checklist for interviews and focus groups. International Journal for Quality in Health Care. 2007. Volume 19, Number 6: pp. 349 – 357.*
