# Supplementary material for: Creating work-life balance among physicians in the age of digitalization: the role of self-consciousness and communication – a qualitative study
Source: BMC Health Serv Res. 2023 Oct 24;23:1141. doi: 10.1186/s12913-023-10101-w (PMC10594792; doi:10.1186/s12913-023-10101-w)
Supplement: Supplementary file 2 — Supplementary Material 2 [file 12913_2023_10101_MOESM2_ESM.docx]

**INTERVIEW GUIDE**

1. **Introduction**

Thank you for agreeing to participate in this interview! Please introduce yourself briefly.

(Please include the following information: age, gender, place of residence, qualification(s), type of current job (private, public...), position, private practice, sideline)

**2. Work and career choice (warm-up questions, a few minutes)**

First, I would like to ask you about your career and your current job.

- How and when did you choose to become a doctor?
- Tell us about your current job (what is your speciality, what are the daily challenges).
- What major changes in your profession/work have you experienced in your career so far?

**3. Technological changes**

Over the past decades, the use of digital technologies such as the internet and mobile phones has significantly changed people's health-related habits and behaviour.

- What is your first association with the term digital health?
- What role do modern technological devices e.g. computer, tablet, mobile phone, smart watch etc. play in your work?
- What digital tools do you currently use in your work (computer, tablet, mobile phone, smart watch, sensors, apps, etc.)?

*Facilitating questions when he/she doesn't really know what to think about:*

*Do you:*

- *communicate online with patients?*
- *share medical records digitally?*
- *Skype, video chat with a patient?*
- *use social media (FB) to communicate with the patient?*
- *make appointments online?*
- *look up medical information online?*
- *write an e-prescription?*
- *use or recommend health monitoring mobile apps to patients?*
- *use health monitoring sensors?*
- *use portable diagnostic tools?*
- *access professional materials (literature, conferences...etc)?*
- Which of these can you use effectively? What do you find useful?
- What changes have you experienced in this area during the Covid epidemic?
- What do you want to keep from the changes? What would you like to leave as soon as possible?
- What digital solutions would you like to try? What are the barriers to trying/using it if you don't succeed?
- What are the advantages and disadvantages of using information technology? (from a professional point of view, from a time management point of view, from a patient relations point of view, from a work-life point of view)

**4. Doctor-patient relationship**

- How do you see the doctor-patient relationship changing since you started practising?
- What factors do you see as the reasons for the changes?
- Do you think the doctor-patient relationship has changed with the use of digital health? If so, how is it changing? What advantages or disadvantages do you see?
- How digitally prepared do you feel your patients are? How prepared do you feel yourself?
- How open do you feel your patients are to using digital health?
- How open are you to using digital tools?
- How open do you feel your colleagues are about to using digital tools?

**5. Summary and looking to the future**

- What changes do you think digital health will bring to doctors' lives?
- How do you think the development of digital technology will affect the healthcare system? And the doctor-patient relationship?
- What implications will these changes have for the skills needed by doctors?

**6. Closing**

- Is there anything else you would like to share on this topic?
